# A Rare Case of an Unroofed Coronary Sinus With a Persistent Left Superior Vena Cava Diagnosed by Two-Dimensional Transthoracic Echocardiography

**DOI:** 10.7759/cureus.13041

**Published:** 2021-01-31

**Authors:** Gajanan Khadkikar, Subrahmanya Murti V, Aman Patel, Sanjay C Shah, Tejas M Patel

**Affiliations:** 1 Cardiology, Smt. Nathiba Hargovandas Lakhmichand (NHL) Municipal Medical College, Ahmedabad, IND; 2 Interventional Cardiology, Apex Heart Institute, Ahmedabad, IND

**Keywords:** coronary sinus, left superior vena cava, echocardiography, atrial septal defect

## Abstract

An unroofed coronary sinus is a rare congenital anomaly in the roof of the coronary sinus causing a communication between the coronary sinus and the left atrium leading to a left to right shunt. It is often associated with a persistent left superior vena cava and other complex congenital lesions like anomalous pulmonary venous return and heterotaxy. Since it is a deep-seated defect, it is seldom diagnosed by transthoracic two-dimensional (2D) echocardiography and requires multimodal imaging for a diagnosis. Here, we present the case of a 27-year-old male in whom the defect was very apparent on standard 2D transthoracic echocardiography. Transthoracic 2D echocardiography revealed situs solitus, levocardia, and a dilated coronary sinus with unroofing which was most prominent in the standard parasternal long-axis view and the foreshortened apical four-chamber view. A color Doppler demonstrated a flow from the left atrium into the dilated coronary sinus. The right ventricle and atrium were dilated with mild pulmonary arterial hypertension. There was no right ventricular dysfunction. Examination with modified suprasternal views showed a left superior vena cava. All four pulmonary veins drained into the left atrium. Other chambers of the heart and great vessels were structurally normal without coarctation or patent ductus arteriosus. The interventricular septum was intact and atrioventricular and ventriculoatrial concordance was preserved. Detection of a dilated coronary sinus by transthoracic 2D echocardiography must be followed by multimodal imaging techniques like cardiac computed tomography and transesophageal echocardiography to detect and manage associated defects.

## Introduction

An unroofed coronary sinus (UCS) is a rare congenital anomaly. Although classified as being a type of atrial septal defect (ASD), it is a communication between the left atrium and the coronary sinus as it courses posterior to the left atrium thereby communicating into the right atrium. It thus physiologically behaves like an ASD and is thought to be the rarest among the ASDs, representing <1% of all cases [1]. Most cases of UCS are associated with a persistent left superior vena cava (PLSVC) and this complex was first described by Raghib et al [2]. UCS may also be associated with other complex cardiac congenital anomalies like anomalous pulmonary venous return and heterotaxy. Like many ASDs, clinical features and electrocardiography (ECG) may be nonspecific. Further, being a deep-seated defect, it is difficult to diagnose on standard transthoracic two-dimensional (2D) echocardiography. It is best diagnosed by multimodality imaging including transesophageal echocardiography and cardiac computed tomography [3]. Here, we present a case wherein the anomaly was clearly apparent on 2D transthoracic echocardiography.

## Case presentation

**Figure 1:**
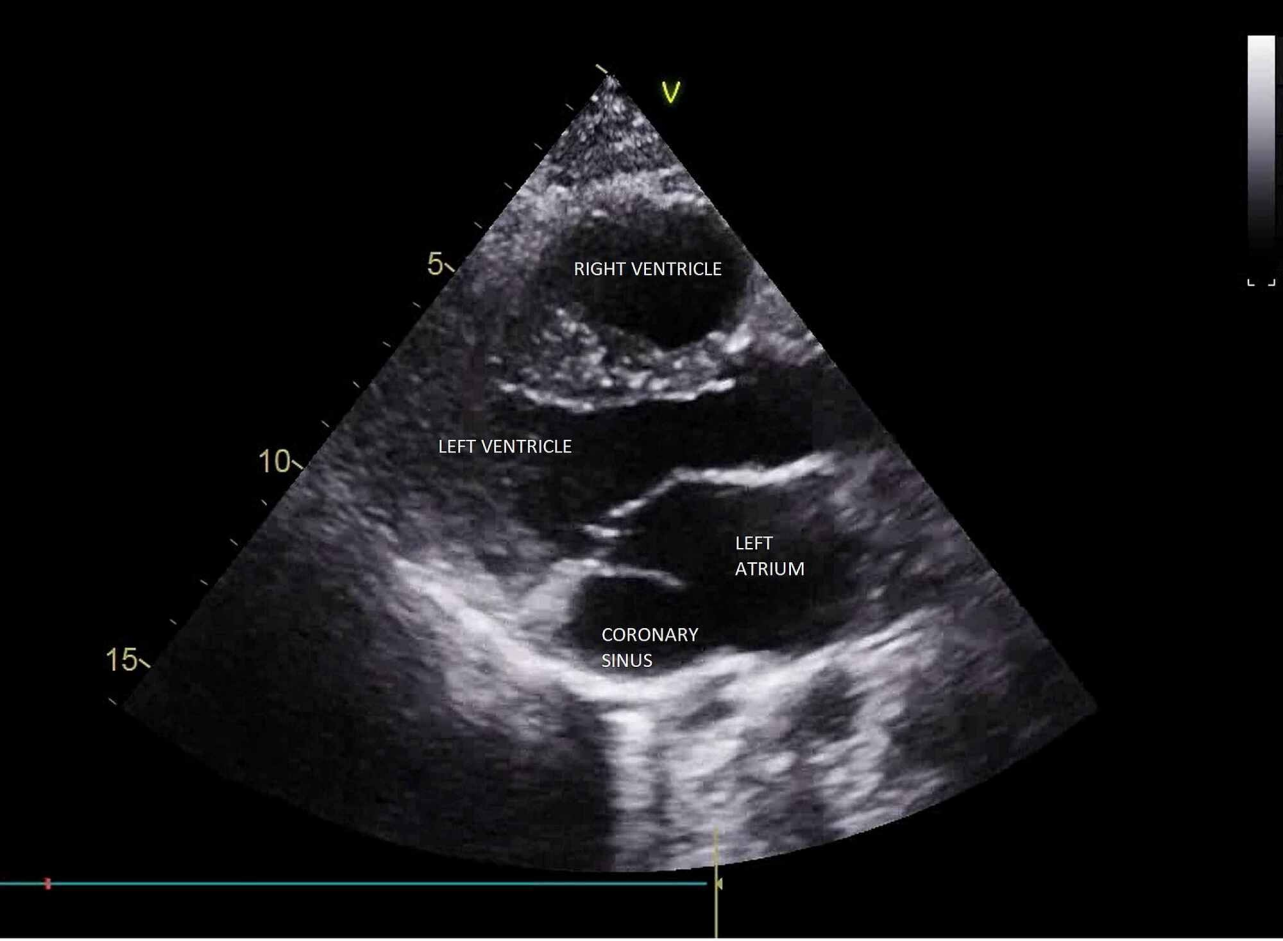
Parasternal long-axis view showing a dilated coronary sinus

**Figure 2:**
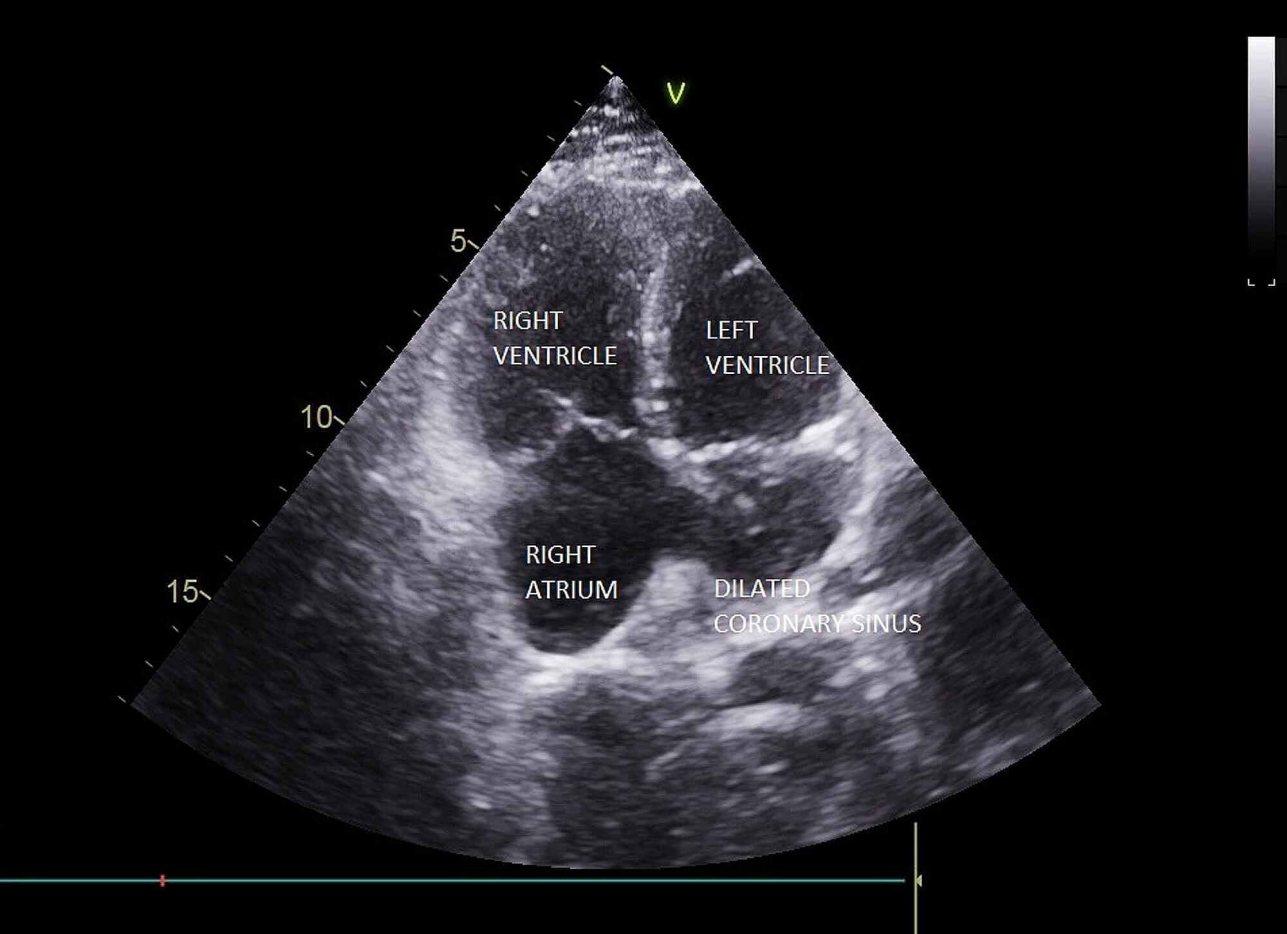
Foreshortened apical four-chamber view showing a dilated coronary sinus

**Figure 3:**
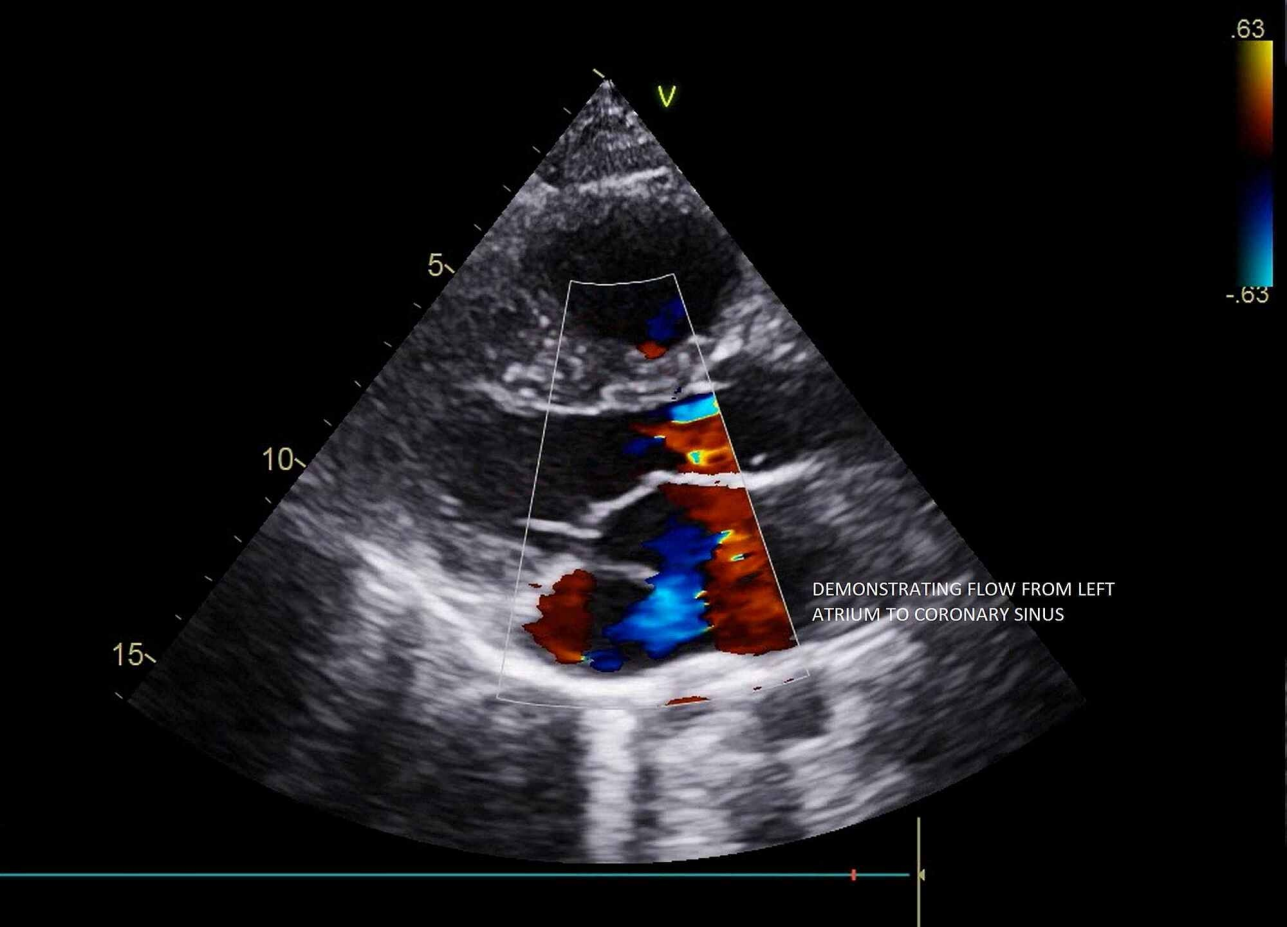
Color Doppler showing flow from the left atrium into the dilated coronary sinus

A 27-year-old asymptomatic male was referred for a cardiac evaluation after a pre-employment electrocardiograph (ECG) showed a right bundle branch with a right ventricular strain pattern. On clinical examination, chest auscultation was normal, cardiac auscultation revealed a fixed-wide split second heart sound without any murmurs. X-Ray of the chest was unremarkable. Transthoracic 2D echocardiography revealed situs solitus, levocardia, and a dilated coronary sinus with unroofing most prominent in the standard parasternal long-axis view (Figure 1, Video 1) and the foreshortened four-chamber view (Figure 2). Color Doppler demonstrated a flow from the left atrium into the dilated coronary sinus (Figure 3, Video 2) demonstrating a left to right shunt.

**Video 1:** Parasternal long-axis view showing a dilated coronary sinus

**Video 2:** Color Doppler on the parasternal long-axis view showing flow from the left atrium into the dilated coronary sinus

**Figure 4:**
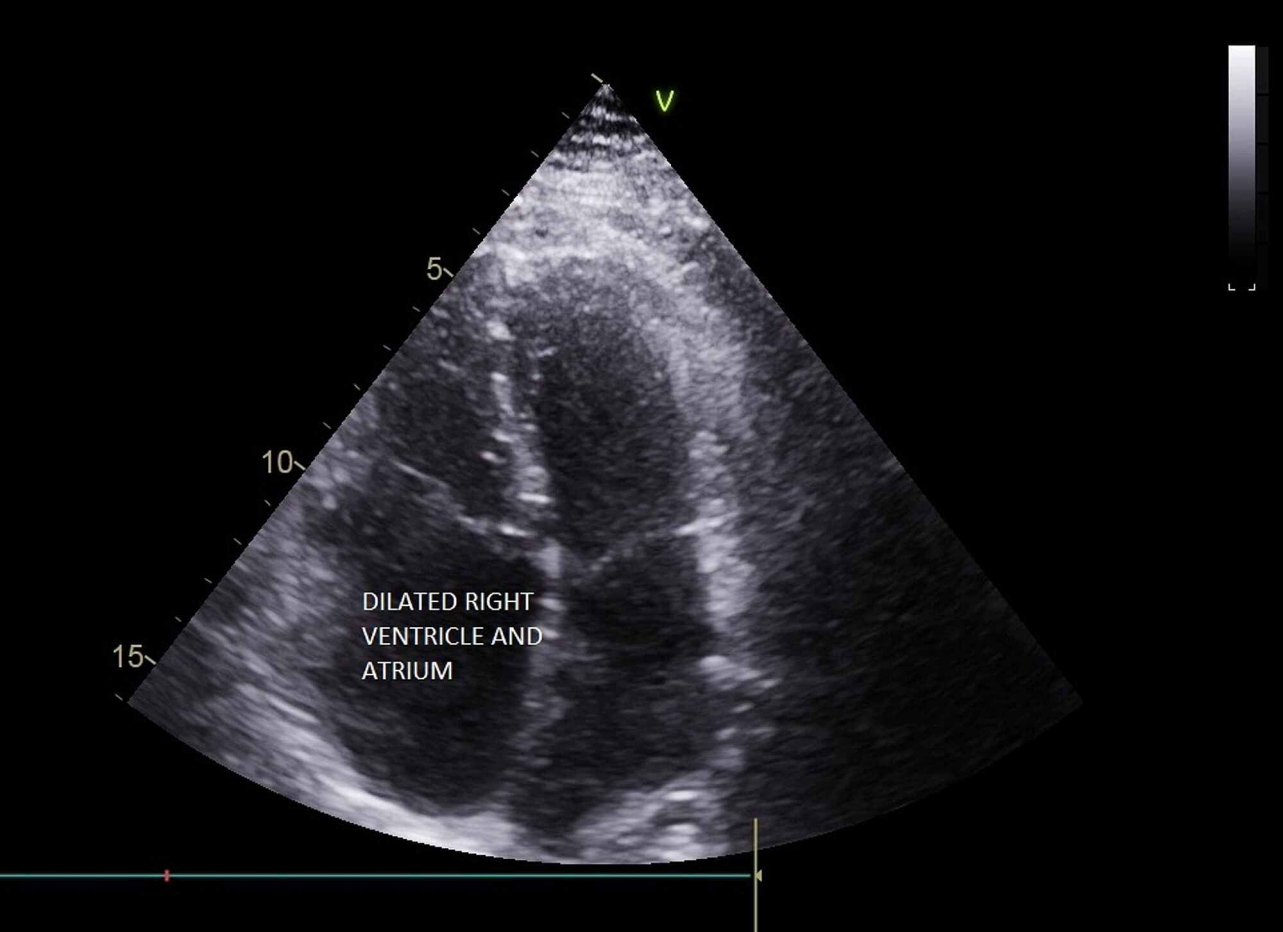
Apical four-chamber view showing dilation of the right atrium and ventricle

**Figure 5:**
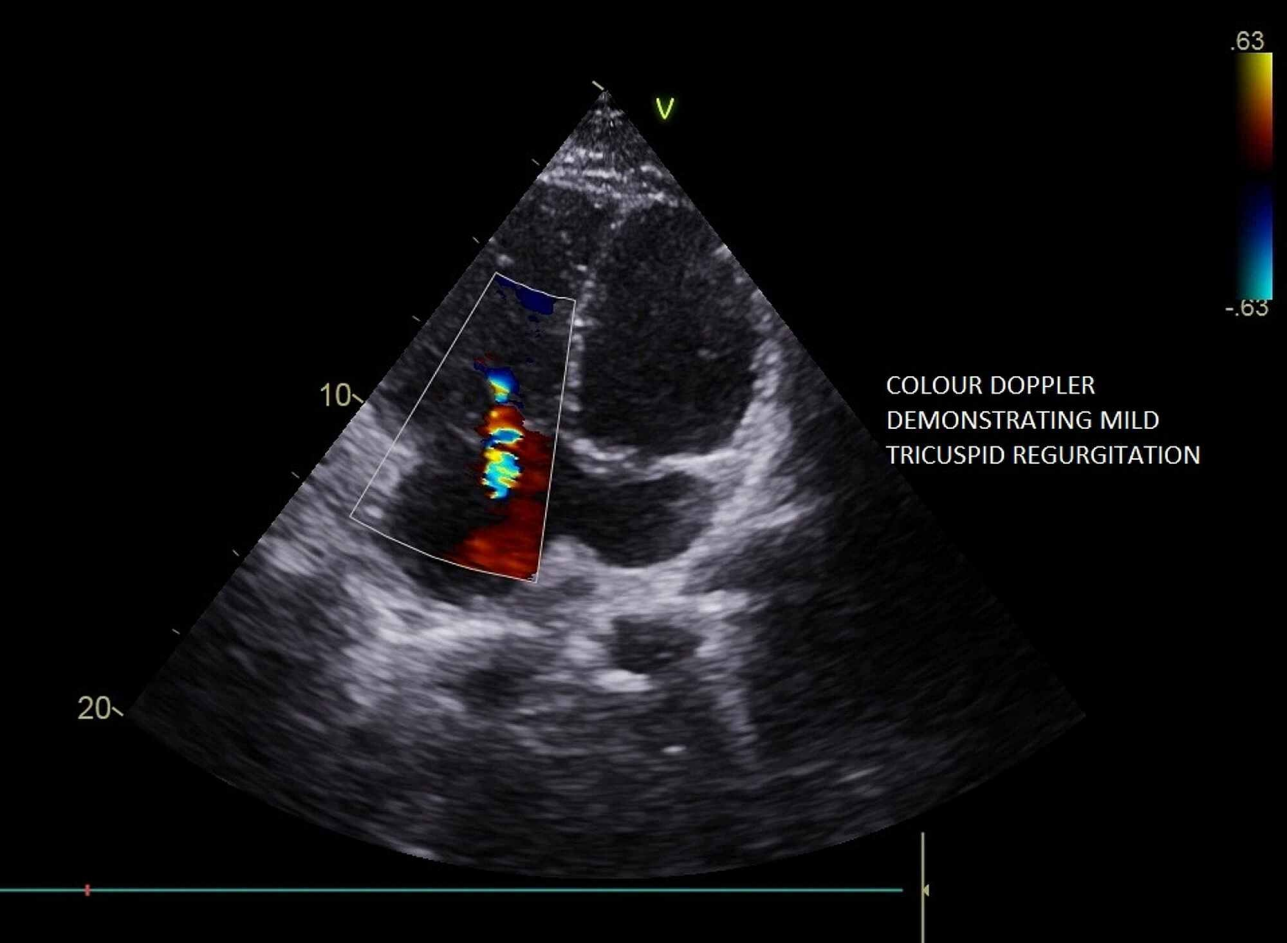
Color Doppler showing mild tricuspid regurgitation

**Figure 6:**
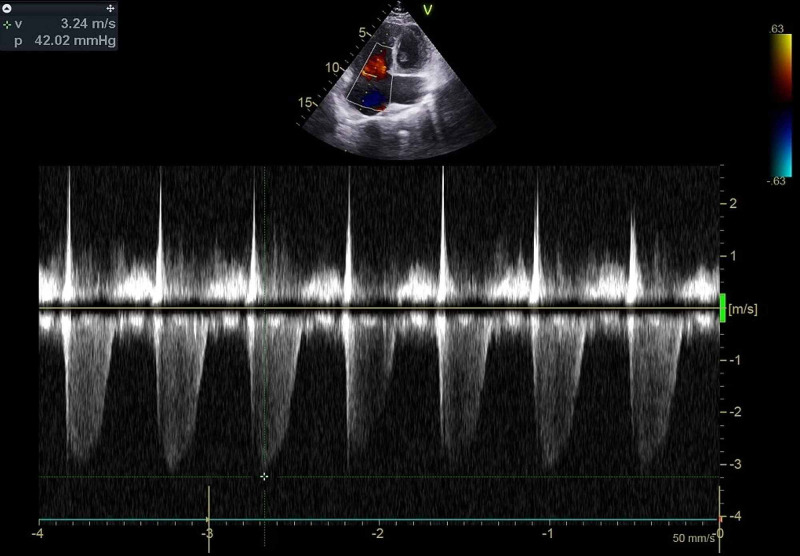
Continuous wave Doppler signal of the tricuspid regurgitation jet

**Figure 7:**
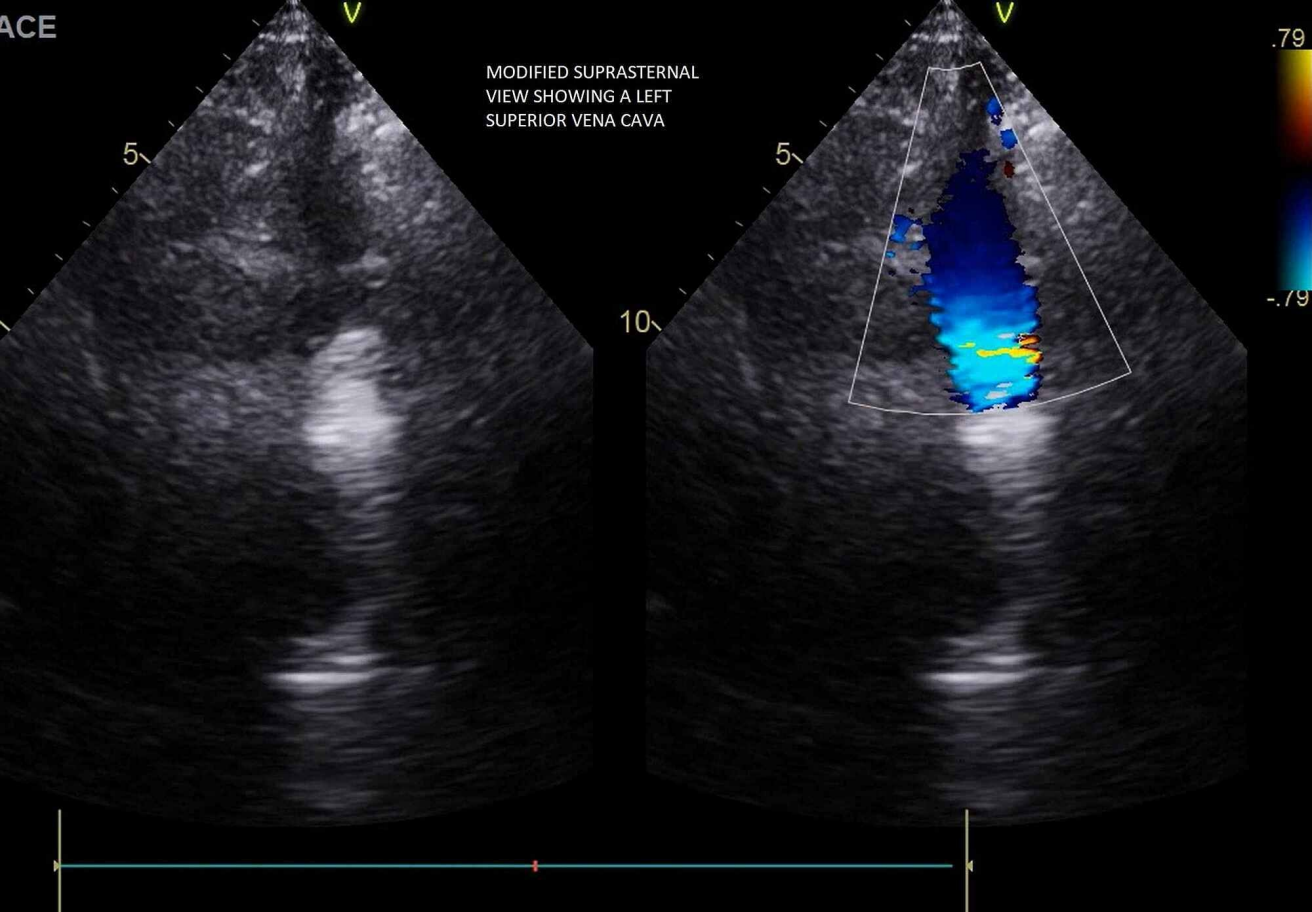
Modified suprasternal view with color Doppler showing a persistent left superior vena cava

**Figure 8:**
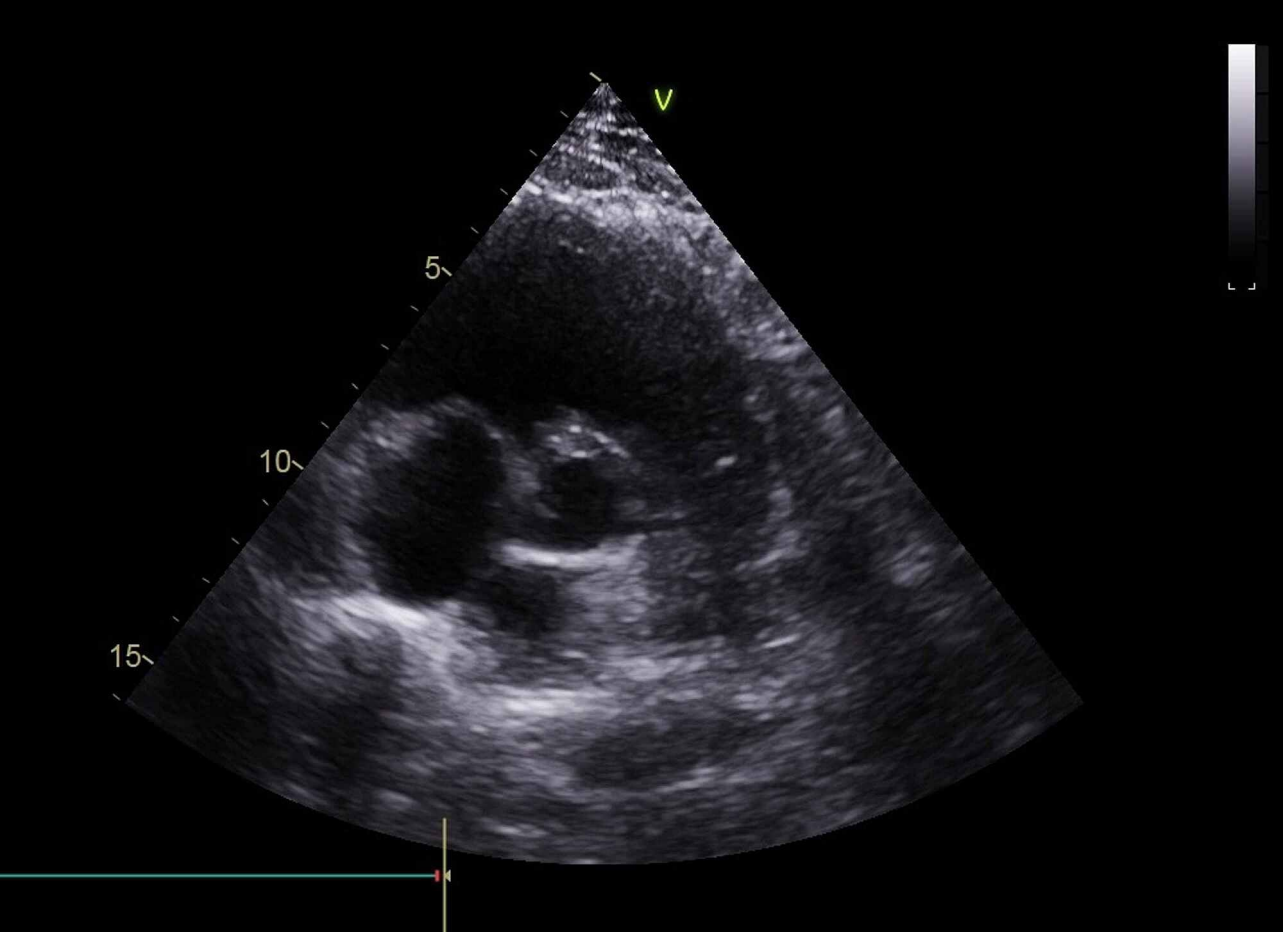
Parasternal short-axis view showing the pulmonary valve and main pulmonary artery

**Figure 9:**
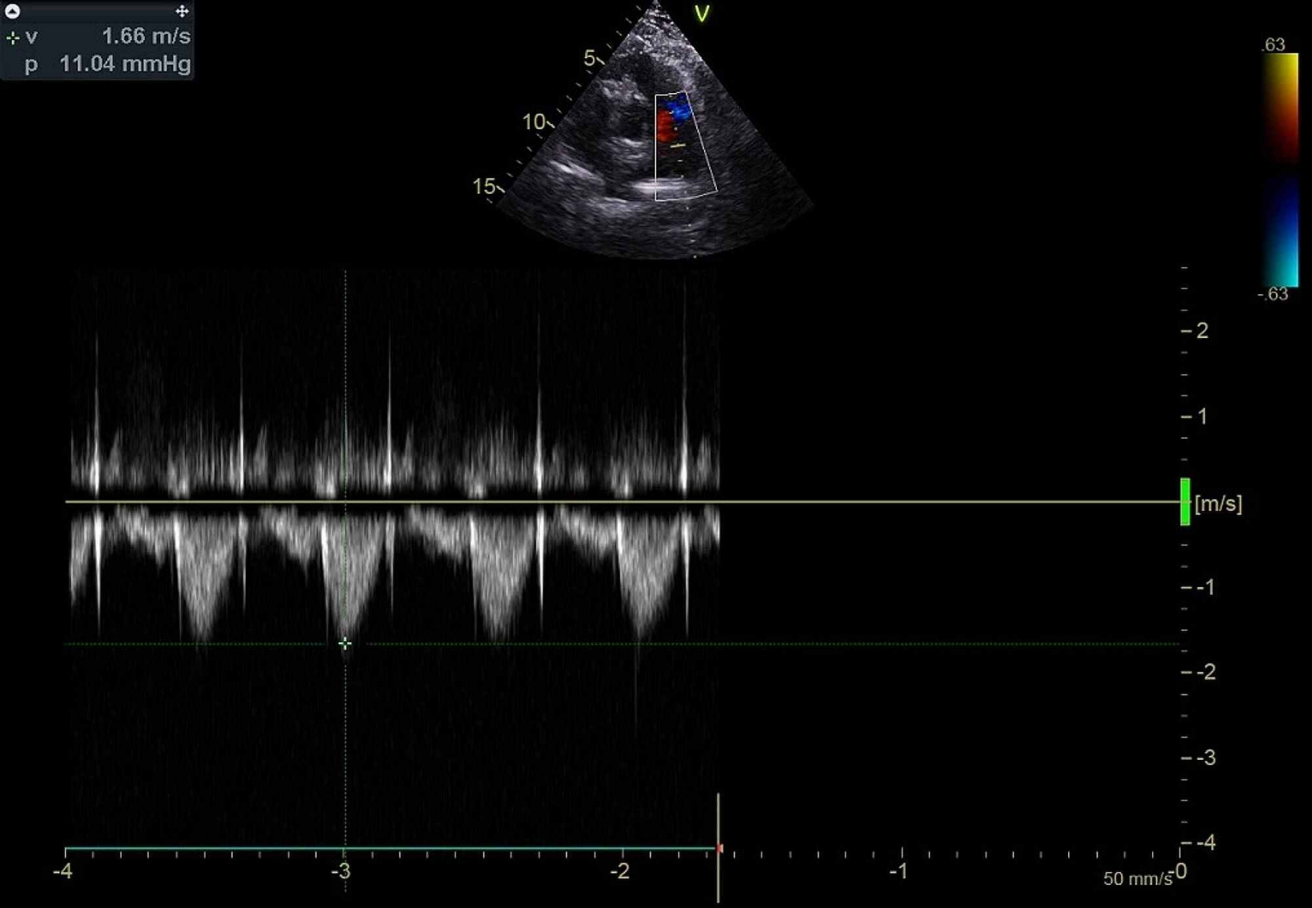
Continuous wave Doppler signals from the pulmonary valve showing normal flow velocity

The right ventricle and atrium were dilated (Figure 4) with mild tricuspid regurgitation (TR) (Figure 5). The calculated right ventricular systolic pressure (RVSP) from the TR jet maximum velocity and right atrial pressure was 47 mmHg, suggesting mild pulmonary arterial hypertension (Figure 6). Examination in modified suprasternal views showed a left superior vena cava (Figure 7, Video 3). There was no right ventricular outflow obstruction, pulmonary stenosis, or patent ductus arteriosus; the pulmonary flow velocity was normal (Figures 8, 9). All four pulmonary veins drained into the left atrium. Other chambers of the heart and great vessels were structurally normal. The aortic arch was left-sided without coarctation. The interventricular septum was intact. Atrio-ventricular and ventriculoatrial concordance were preserved.

**Video 3:** Color Doppler in a modified suprasternal view showing a persistent left superior vena cava

## Discussion

An unroofed coronary sinus presents a double problem as opposed to the more common ostium secundum type of ASDs. Being a left-to-right shunt, there is a predisposition toward right heart failure and pulmonary hypertension. Pulmonary arterial hypertension usually presents late in the disease course but has been reported even as early as six months of age [4]. Further, because of the coronary sinus being connected to the left atrium, part of the venous return may connect to the left, leading to complications from paradoxical emboli including cerebral abscesses [2,5]. Anatomically, Kirklin and Barratt-Boyes classified UCS into four types - Type I: Completely unroofed with PLSVC, Type II: Completely unroofed without PLSVC, Type III: Partially unroofed mid-portion, and Type IV: partially unroofed terminal portion [6]. The present case may likely represent a type IV-like defect.

Transthoracic 2D echocardiography is the first line of investigation to evaluate any ASD including UCS. However, transthoracic echocardiography may be very limited for the diagnosis of UCS since it is a deep-seated lesion. A case series of 23 patients of UCS showed that echocardiography diagnosed only 56.5% of the cases, that too after combining transesophageal echocardiography with transthoracic imaging [7]. Another case series of 20 patients also found a diagnostic accuracy of 65% for echocardiography when contrast transesophageal and transthoracic modes were combined [8]. These works show the difficulty in the diagnosis of UCS with echocardiography. UCS is better diagnosed by computed tomography (CT) and cardiac magnetic resonance imaging (CMR) in combination with echocardiography [9]. An observational study of 23 UCS patients compared cardiac CT angiography to echocardiography and found the diagnostic accuracy of CT imaging to be 100% [10]. Multimodal diagnostic methods should be the standard for diagnosing patients suspected of having UCS to further optimize the eventual surgical correction. The present case was unusual since the anomaly was easily apparent on 2D transthoracic echocardiography. As described above, this is usually not the case and even if a definitive diagnosis is made on echocardiography, a follow-up imaging with cardiac CT or CMR must be done to complete the evaluation.

## Conclusions

A coronary sinus atrial septal defect is a well-described albeit rare congenital anomaly. Transthoracic echocardiography is employed as the first line of imaging for diagnosing the suspected cases since the technique is non-invasive. However, the accuracy of echocardiography in general, and transthoracic echocardiography in particular, for diagnosing UCS is poor. If the presence of a dilated coronary sinus on echocardiography is corroborated by clinical, ECG, and X-ray evaluation, then multimodal imaging techniques must be employed to detect associated atrial septal defects and guide subsequent therapy.
